# Association between flexibility assessment based on prone full-spine CT scout view and surgical strategy for old thoracolumbar fracture with kyphotic deformity

**DOI:** 10.3389/fsurg.2026.1834303

**Published:** 2026-09-22

**Authors:** Xiubo Ge, Yifei Li, Liang Zhao, Haitao Lu, Wei Zhang, Kangkang Wang, Haiyang Yu

**Affiliations:** 1 Department of Orthopedics, Fuyang People's Hospital Affiliated to Anhui Medical University (Fuyang People's Hospital), Fuyang, Anhui, China; 2 Department of Orthopedics, Fuyang People's Hospital of Bengbu Medical University (Fuyang People's Hospital), Fuyang, Anhui, China; 3 Clinical Research Center for Spinal Deformity of Anhui Province, Fuyang, Anhui, China

**Keywords:** deformity, FLS-CT, fusion, osteotomy, position, spinal flexibility, surgical planning

## Abstract

**Objective:**

This study aimed to investigate whether spinal flexibility, assessed by the alignment difference between Full-length Spine CT scout view (FLS-CT) and standing radiographs, was associated with the eventual use of three-column osteotomy (3CO) in patients with old thoracolumbar fracture with kyphotic deformity (OTLFK).

**Methods:**

This single-center, dual-surgeon retrospective study included patients with OTLFK who underwent corrective surgery between August 2021 and August 2024. Patients were categorized into the 3CO group and the non-three-column osteotomy (N3CO) group according to whether 3CO was ultimately performed. Demographic characteristics, surgical variables, radiographic parameters, and clinical outcomes were compared between groups. Clinical outcomes included Visual Analog Scale (VAS) and Oswestry Disability Index (ODI) assessed at 1 month, 6 months, and final follow-up. To quantify positional flexibility relative to the correction ultimately achieved, the kyphosis flexibility percentage (Kflx%) was retrospectively calculated using local kyphosis Cobb angle (LKCA) measurements from preoperative standing radiographs, preoperative prone FLS-CT, and postoperative standing radiographs. Because its denominator included postoperative standing LKCA, Kflx% was treated as a postoperative target-referenced metric rather than a purely preoperative predictor. Its apparent discrimination for classifying the observed use of 3CO was explored using a receiver operating characteristic (ROC) curve.

**Results:**

A total of 109 patients were included, comprising 75 in the 3CO group and 34 in the N3CO group. Compared with the N3CO group, the 3CO group had significantly lower preoperative standing lumbar lordosis and significantly higher preoperative prone LKCA and T1 pelvic angle. Kflx% was significantly lower in the 3CO group than in the N3CO group (34.2% vs. 60.6%, *P* < 0.001). Among the variables entered into the multivariate logistic regression model, Kflx% was independently associated with the observed use of 3CO (OR = 0.940, 95% CI 0.910–0.970, *P* < 0.001). ROC analysis identified an exploratory, cohort-derived Kflx% threshold of 49.7%, with a sensitivity of 82.7%, specificity of 61.8%, and positive predictive value of 82.9% for classifying patients who ultimately underwent 3CO. VAS and ODI improved significantly after surgery in both groups, without significant between-group differences at the same follow-up time points.

**Conclusion:**

In this institutional cohort, lower Kflx% was associated with 3CO use. Kflx% was calculated retrospectively and normalized to the correction achieved after surgery. The 49.7% cutoff is exploratory and should not be regarded as a validated preoperative decision threshold. Prospective evaluation using alignment targets defined before surgery is required.

## 1 Introduction

Old thoracolumbar fracture with kyphotic deformity (OTLFK) often results from improper or delayed treatment of thoracolumbar fractures and usually requires corrective osteotomy (1–3). With the growing understanding of spinal biomechanics, restoration of optimal sagittal alignment has become the primary surgical objective, leading to an increased application of three-column osteotomy (3CO), particularly pedicle subtraction osteotomy (PSO, Grade III) and modified pedicle subtraction osteotomy (mPSO, Grade IV) (4–6). Higher-grade osteotomies involve greater bone resection, more extensive trauma, increased blood loss, and longer operative time, and carry higher risks of spinal cord or nerve root injury and cord shortening (7–9). In some elderly patients, these factors may preclude the possibility of revision surgery. However, not all severe kyphotic deformities require 3CO to achieve satisfactory correction, as the relative flexibility of the deformity often determines whether alignment goals can be reached without such extensive procedures (10, 11). Accurate preoperative estimation of osteotomy grade is therefore important for reducing complications and optimizing surgical strategy.

Static standing radiographs have limitations in assessing deformity flexibility, which is a key factor in determining the necessity and type of osteotomy (12). The differences between standing and supine or sitting images can reveal spinal deformability under different postures, allowing quantitative assessment of curvature flexibility and guiding individualized surgical planning (13–15). Inspired by this concept, our team proposed the use of the Full-length Spine CT scout view (FLS-CT) to simulate the intraoperative posture (16). In this method, the patient is positioned prone with positioning pads placed under the chest and anterior superior iliac spines, allowing gravity to relax the paraspinal muscles and reveal the true spinal contour. The angular change in local kyphosis caused by postural adjustment is then quantified to evaluate sagittal kyphosis flexibility. Previous studies have demonstrated that FLS-CT not only predicts the actual intraoperative kyphotic angle and assists in surgical planning but also helps anticipate proximal junctional kyphosis following adult spinal deformity correction (17, 18). Therefore, this study aimed to investigate whether spinal flexibility, defined by the difference between prone and standing images, was associated with the eventual use of 3CO in OTLFK.

## 2 Materials and methods

### 2.1 Study design and patient selection

We conducted a single-center, dual-surgeon retrospective analysis of patients with OTLFK who underwent corrective surgery between August 2021 and August 2024. Inclusion criteria: ① Definite diagnosis of OTLFK with significant low back pain or lower limb symptoms due to spinal cord or nerve compression, and ineffective conservative treatment for ≥3 months; ② Multilevel fusion involving ≥4 segments; ③ Complete preoperative and postoperative imaging data, including preoperative standing radiographs, preoperative FLS-CT, and postoperative standing radiographs at 1 month. Exclusion criteria: ① Degenerative or congenital kyphotic deformity; ② Poor general condition precluding surgery; ③ Incomplete imaging data. This study was approved by the institutional review board (approval No. [2024]223).

### 2.2 Data collection

Demographic characteristics and surgical data were collected from all patients. The surgical data included the number of fused segments and intraoperative blood loss. Osteotomy procedures were recorded and standardized according to the classification system proposed by Schwab et al. (19). Standing full-spine x-rays and FLS-CT were obtained preoperatively. Standing x-rays were collected at the one-month postoperative follow-up. VAS and ODI were assessed before surgery and at 1 month, 6 months, and final follow-up. Follow-up duration was defined as the interval between surgery and the final clinical assessment.

Both treating surgeons had access to the preoperative FLS-CT images as part of the routine imaging assessment. Changes in spinal alignment between the standing and prone positions were considered during surgical planning. The surgeons also assessed deformity severity and neurological status. Bone quality and the intended correction were also considered. Intraoperative findings could further influence the final osteotomy grade.

However, the Kflx% examined in this study was not available during surgical decision-making. Its calculation included postoperative standing LKCA. Therefore, Kflx% and the 49.7% cutoff were derived retrospectively. No formal cutoff based on Kflx% was used to determine whether 3CO should be performed. Nevertheless, the surgeons reviewed the FLS-CT findings before surgery. Therefore, potential incorporation bias between the imaging findings and the surgical decision cannot be excluded.

### 2.3 Imaging examination method

① Standing full-spine lateral radiographs: Patients stood erect naturally without support, with arms flexed and fingers touching the clavicles to avoid upper limb overlap with the spinal image (20). ② FLS-CT: A 128-slice spiral CT scanner (Neusoft Medical Systems Co., Ltd.) was employed, with tube voltage of 120 kV and tube current of 250 mA. Scanning was conducted jointly by one spine surgeon and one radiologist. The patient was positioned prone on the CT table, with specialized pads (45 cm × 15 cm × 12 cm) placed beneath the sternum and bilateral anterior superior iliac spines, suspending the lower chest and abdomen. Upper limbs were flexed naturally beside the head and neck, with the head in a horseshoe headrest to maintain cervical neutrality. Following adaptation, paraspinal muscles relaxed, allowing gravity-induced sagging of the chest and abdomen, traction on the thoracolumbar spine, and spontaneous kyphosis reduction. A full-spine CT scout view was then acquired from skull to pelvis. See Figure 1.

**Figure 1:**
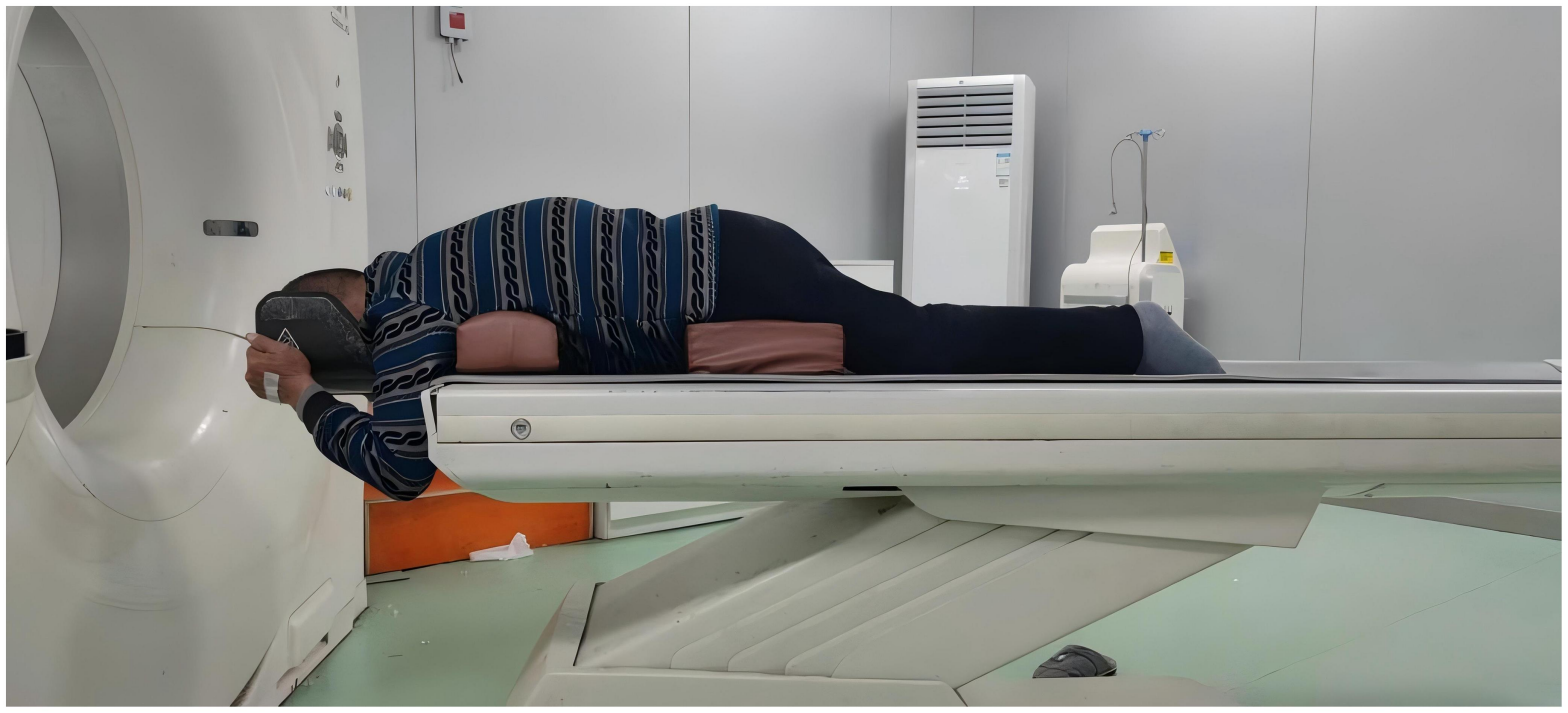
Schematic illustration of patient positioning for full-length spine CT scout view (FLS-CT).

### 2.4 Radiographic parameters

Preoperative and one-month postoperative standing x-ray and FLS-CT data were imported into Surgimap software (Nemaris Inc., USA) to obtain sagittal spinal profiles. Two experienced spine surgeons independently measured the following parameters, and the mean values were used for analysis: sagittal vertical axis (SVA), thoracic kyphosis (TK), local kyphosis Cobb angle (LKCA, defined as the angle between the superior endplate of the vertebra above and the inferior endplate of the vertebra below the fractured segment), pelvic tilt (PT), pelvic incidence (PI), lumbar lordosis (LL), PI–LL mismatch, and T1 pelvic angle (TPA).

### 2.5 Statistical analysis

Patients were classified according to whether a three-column osteotomy was performed: those who underwent 3CO were assigned to the three-column osteotomy group (3CO group), and those who did not were placed in the non-three-column osteotomy group (N3CO group). To assess spinal kyphosis flexibility, differences in sagittal parameters among preoperative standing, preoperative prone, and postoperative standing images were analyzed for all patients. Previous studies have shown that the alignment observed on FLS-CT effectively simulates the prone position on a Jackson surgical table. The sagittal kyphosis flexibility percentage (Kflx%) was retrospectively defined as: Kflx% = [(Preoperative standing LKCA)−(Preoperative prone LKCA)]/[(Preoperative standing LKCA)−(Postoperative reference standing LKCA)] × 100%. The numerator represents positional reduction from standing to prone imaging, whereas the denominator represents the correction ultimately observed at the one-month postoperative assessment. Because the denominator was unavailable before surgery, Kflx% is a postoperative target-referenced metric and not a fully preoperative standalone predictor. For example, with a preoperative standing LKCA of 60°, a prone LKCA of 35°, and a postoperative reference LKCA of 10°, Kflx% = (60°−35°)/(60°−10°) × 100% = 50%. Thus, the positional change accounted for 50% of the LKCA correction ultimately observed; this retrospective proportion does not by itself determine which procedure was required. Comparisons between the two groups included age, body mass index (BMI), number of fused segments, intraoperative blood loss, bone mineral density (T-score), and radiographic parameters (SVA, TK, LKCA, PT, PI, LL, PI–LL mismatch, and TPA).

All data were analyzed using IBM SPSS Statistics 22.0 (IBM Corp.). Continuous variables were first tested for normality. Variables with a normal distribution were compared using the Student's *t*-test, while those with a non-normal distribution were analyzed with the Mann–Whitney U test. Multivariate logistic regression analysis was performed to examine factors independently associated with the eventual use of three-column osteotomy. Receiver operating characteristic (ROC) analysis was subsequently used to describe the apparent discrimination of Kflx% for the observed use of 3CO and to derive an exploratory within-cohort cutoff, along with its sensitivity and specificity. Because the cutoff was derived and evaluated in the same retrospective cohort, it was not interpreted as a validated prospective decision threshold. A *p*-value < 0.05 was considered statistically significant.

## 3 Results

### 3.1 Baseline characteristics

A total of 109 patients were included in this study, comprising 75 (68.9%) who underwent three-column osteotomy (3CO) and 34 (31.2%) who received posterior column osteotomy or non-osteotomy fusion fixation. The majority of patients were female (79.8%), with a mean age of 64.4 ± 7.1 years, a mean BMI of 24.9 ± 3.8 kg/m^2^, and a mean bone mineral density T-score of −3.0 ± 0.9. No significant differences were observed between the two groups in terms of age, sex, BMI, bone mineral density T-score, or number of fused segments (*p* > 0.05; Table 1). However, intraoperative blood loss was significantly higher in the 3CO group compared with the N3CO group (*p* < 0.05).

**Table 1:** Demographic and surgical characteristics of the study population.

Baseline data	N3CO group(*n* = 34)	3CO group(*n* = 75)	t/*χ*^2^/Z	P
Demographic Data				
Sex (male/female, n)	8/26	14/61	0.343	0.558
Age (years)	64.6 ± 7.3	64.3 ± 7.1	0.193	0.847
BMI (kg/m^2^)	25.6 ± 4.2	24.6 ± 3.7	1.274	0.206
Bone Mineral Density (T-score)	−3.1 ± 0.8	−3.0 ± 0.9	−0.490	0.625
Surgical Data				
Number of Fused Segments [M (Q1, Q3), n]	6 (5,7)	7 (5,8)	−1.563	0.118
Intraoperative Blood Loss [M (Q1, Q3), mL]	700 (300,1125)	1,000 (800,1600)	−3.712	**<0**.**001**

Bold values indicate statistically significant differences (*P* < 0.05).

### 3.2 Radiographic analysis

As shown in Table 2, there were no significant differences between the two groups in preoperative standing parameters, including SVA (*P* = 0.728), TK (*P* = 0.255), LKCA (*P* = 0.834), PT (*P* = 0.085), PI (*P* = 0.538), PI–LL mismatch (*P* = 0.186), and TPA (*P* = 0.329). In contrast, the preoperative standing LL was significantly lower in the 3CO group (35.0 ± 14.8°) compared with the N3CO group (41.4 ± 14.2°) (*P* = 0.038). The preoperative prone LKCA was 32.5 ± 12.2° in the 3CO group and 25.3 ± 9.2° in the N3CO group (*P* = 0.003). Similarly, the preoperative prone TPA was significantly higher in the 3CO group (21.9 ± 7.6°) than in the N3CO group (18.5 ± 9.5°) (*P* = 0.047).

**Table 2:** Comparison of radiographic parameters between the Two groups (±SD).

Parameter	N3CO group(*n* = 34)	3CO group(*n* = 75)	P
Preop Standing			
SVA(mm)	30.8 ± 56.8	34.7 ± 52.7	0.728
TK(°)	40.5 ± 19.3	36.2 ± 17.8	0.255
LKCA(°)	44.5 ± 10.3	43.9 ± 15.6	0.834
PT(°)	22.6 ± 11.4	26.9 ± 12.2	0.085
PI(°)	55.3 ± 15.1	53.5 ± 13.9	0.538
LL(°)	41.4 ± 14.2	35.0 ± 14.8	**0**.**038**
PI-LL(°)	14.0 ± 16.4	18.5 ± 16.4	0.186
TPA(°)	30.0 ± 10.0	32.5 ± 13.2	0.329
Preop Prone			
LKCA(°)	25.3 ± 9.2	32.5 ± 12.2	**0**.**003**
TPA(°)	18.5 ± 9.5	21.9 ± 7.6	**0**.**047**
Postop Standing			
SVA(mm)	21.0 ± 41.8	16.5 ± 45.4	0.623
TK(°)	31.8 ± 14.4	26.8 ± 11.6	0.053
LKCA(°)	13.1 ± 6.9	11.8 ± 6.4	0.350
LL(°)	47.9 ± 10.3	45.8 ± 10.9	0.340
PT(°)	16.5 ± 9.3	17.4 ± 9.5	0.663
PI-LL(°)	7.4 ± 13.3	7.7 ± 14.0	0.918
TPA(°)	17.5 ± 7.6	17.5 ± 8.8	0.984
Kflx%	60.6 ± 24.0	34.2 ± 16.5	**<0**.**001**

Bold values indicate statistically significant differences (*P* < 0.05).

Postoperatively, there were no significant differences between the two groups in SVA (*P* = 0.623), TK (*P* = 0.053), LKCA (*P* = 0.350), LL (*P* = 0.340), PT (*P* = 0.663), PI–LL mismatch (*P* = 0.918), or TPA (*P* = 0.984). Overall, sagittal alignment parameters improved significantly after surgery in all patients (Figure 2). Specifically, in the N3CO group, preoperative values of SVA (30.8 mm), TK (40.5°), LKCA (44.5°), LL (41.4°), PT (22.6°), and PI–LL (14.0°) improved postoperatively to 21.0 mm, 31.8°, 13.1°, 47.9°, 16.5°, and 7.4°, respectively (all *p* < 0.05). Similarly, in the 3CO group, preoperative values of SVA (34.7 mm), TK (36.2°), LKCA (43.9°), LL (35.0°), PT (26.9°), and PI–LL (18.8°) improved postoperatively to 16.5 mm, 26.8°, 11.8°, 45.8°, 17.4°, and 7.7°, respectively (all *p* < 0.05). The magnitude of radiographic improvement did not differ significantly between the two groups.

**Figure 2:**
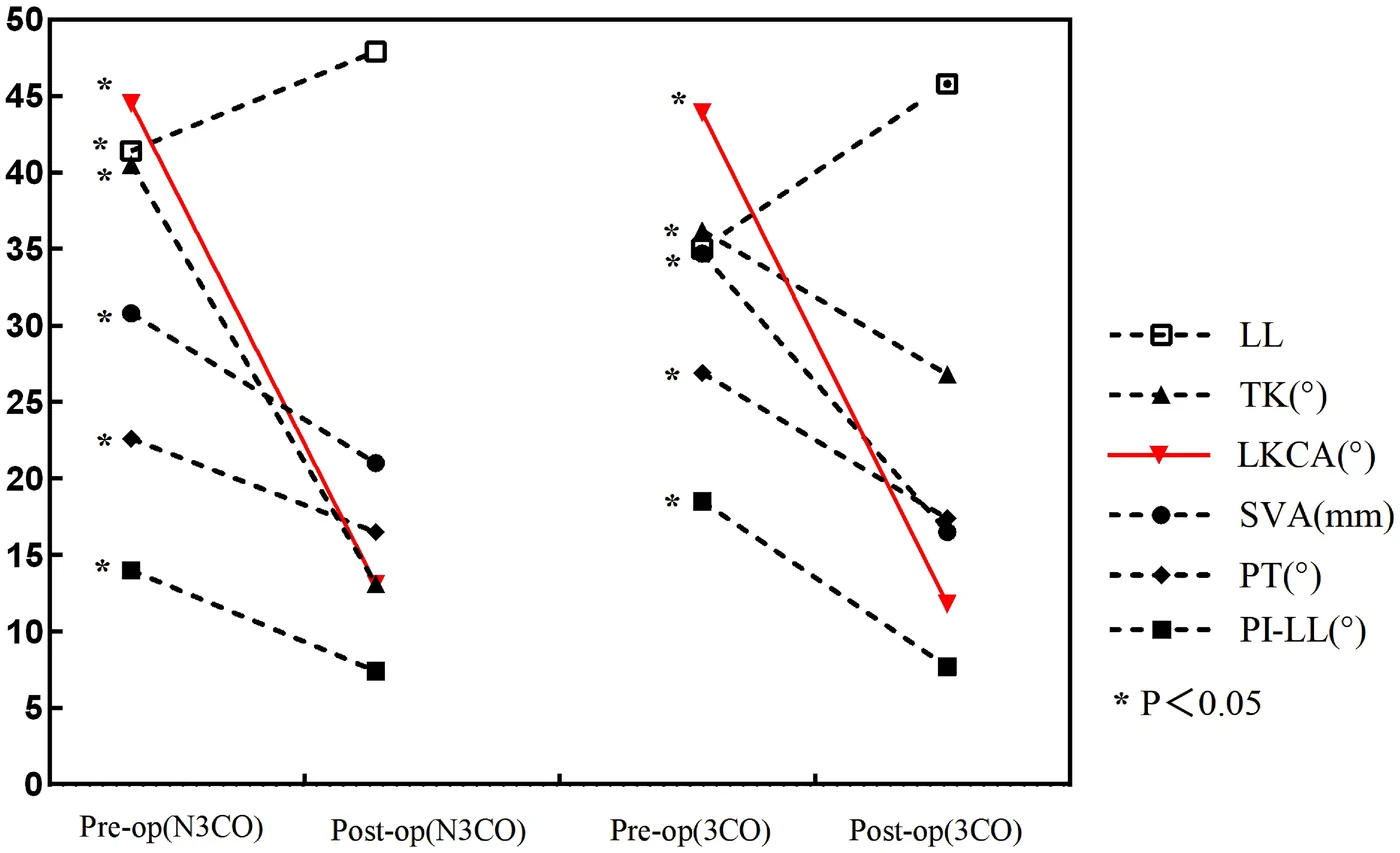
Preoperative and postoperative radiographic findings in the Two groups.

The retrospectively calculated proportion of the observed LKCA correction attributable to positional change (Kflx%) was significantly lower in the 3CO group than in the N3CO group (34.2% vs. 60.6%, *P* < 0.001) (Table 2; Figure 3). Among the variables entered into the multivariate logistic regression model, Kflx% was independently associated with the observed use of three-column osteotomy (*P* < 0.001, OR = 0.940, 95% CI: 0.910–0.970) (Table 3). ROC curve analysis identified an exploratory within-cohort cutoff value of 49.7%. At this threshold, Kflx% showed a sensitivity of 82.7%, specificity of 61.8%, and positive predictive value of 82.9% for classifying patients who ultimately underwent 3CO (Table 4; Figure 4).

**Figure 3:**
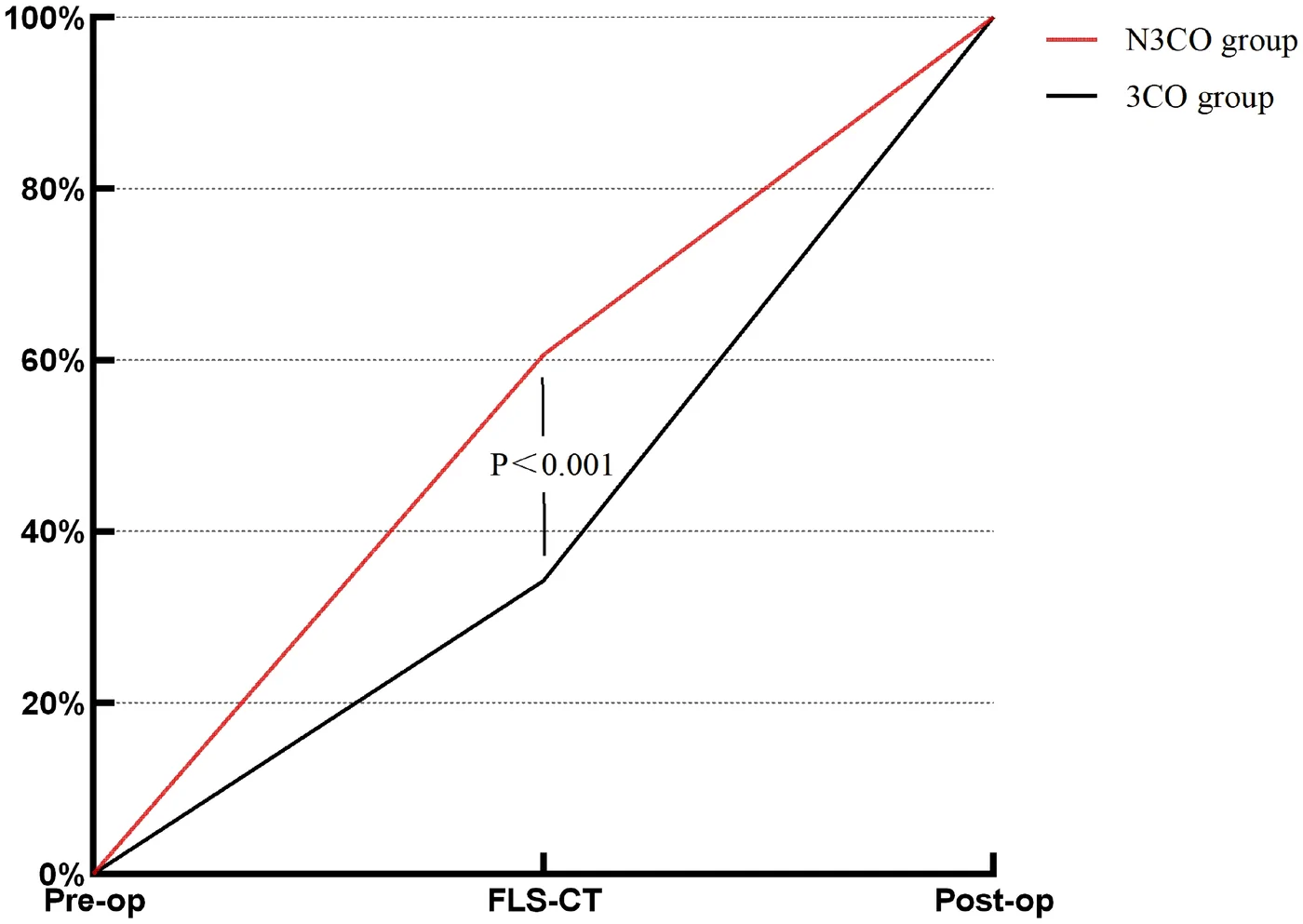
Proportion of LKCA correction (kflx%) derived from FLS-CT and postoperative reference LKCA.

**Table 3:** Multivariate logistic regression analysis of factors associated with the Use of three-column osteotomy.

Variable	*β*	OR	95% CI	P
Preop Standing LL (°)	−0.016	0.984	0.948–1.021	0.400
Preop Prone LKCA(°)	0.019	1.019	0.958–1.084	0.546
Preop Prone TPA(°)	0.055	1.057	0.989–1.129	0.104
Kflx%(%)	−0.062	0.940	0.910–0.970	**<0**.**001**

Bold values indicate statistically significant variables (*P* < 0.05).

**Table 4:** Discriminative performance of kflx% for the eventual Use of three-column osteotomy.

Cut-off value (%)	AUC	Sensitivity (%)	Specificity (%)	95% CI
49.7	0.803	82.7	61.8	0.710–0.896

Bold values indicate statistically significant variables (*P* < 0.05).

**Figure 4:**
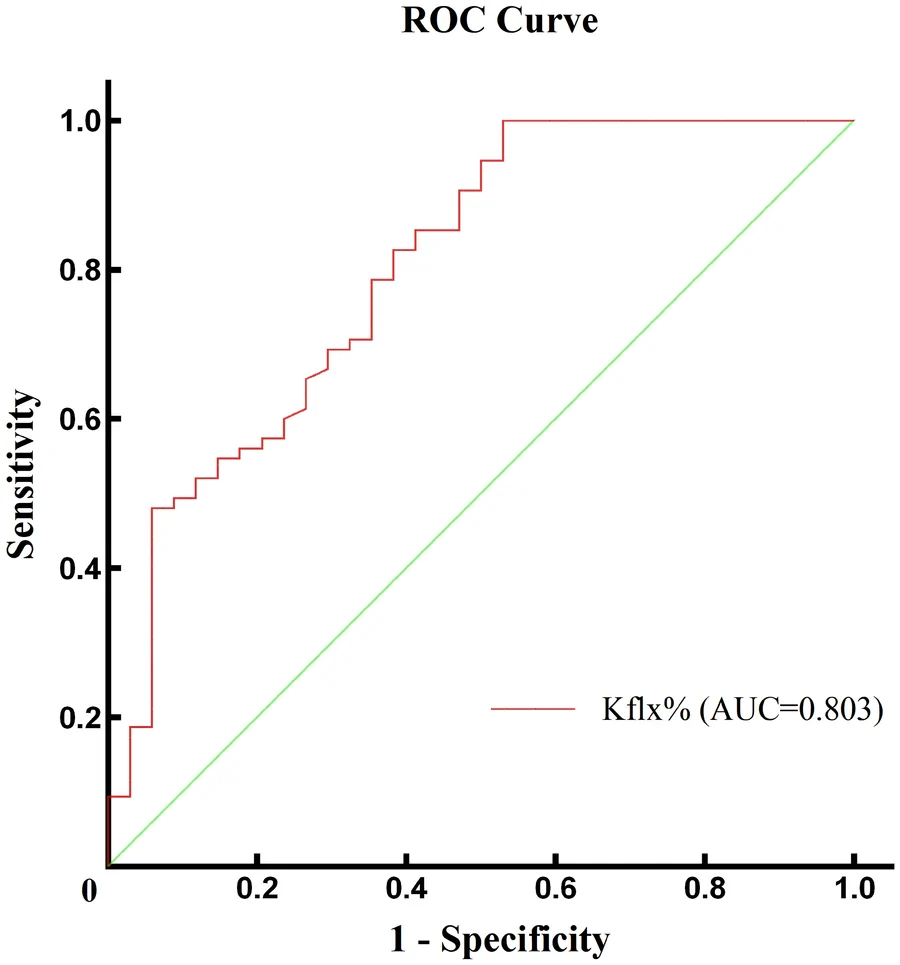
ROC curve of kflx% for discriminating patients who ultimately underwent three-column osteotomy. The area under the curve (AUC) was 0.803 (95% CI: 0.710–0.896). An exploratory cutoff value of 49.7% yielded a sensitivity of 82.7% and a specificity of 61.8%.

### 3.3 Clinical outcomes

The overall mean follow-up was 24.0 ± 1.4 months (range, 20–28 months). It was 24.0 ± 1.0 months in the N3CO group and 24.0 ± 1.5 months in the 3CO group. All 109 patients completed the 1-month, 6-month, and final assessments.

Compared with the preoperative values, VAS scores and ODI at each postoperative time point were significantly decreased in both groups (*P* < 0.05). No significant differences were found in VAS scores or ODI between the two groups at the same time point (*P* > 0.05) (Table 5).

**Table 5:** Comparison of clinical outcomes between the groups.

Variable	N3CO group(*n* = 34)	3CO group(*n* = 75)	P
Visual Analog Scale (VAS)			
Preoperative	6.9 ± 1.0	6.9 ± 1.1	0.898
VAS at 1 month*	2.2 ± 0.9	2.2 ± 1.0	0.901
VAS at 6 months*	1.6 ± 0.6	1.7 ± 0.6	0.713
VAS at final follow-up*	1.4 ± 0.5	1.4 ± 0.5	0.841
Oswestry Disability Index (ODI)			
Preoperative (%)	72.8 ± 3.3	75.6 ± 3.4	0.457
ODI at 1 month (%)*	25.8 ± 2.1	26.4 ± 2.4	0.734
ODI at 6 months (%)*	20.2 ± 2.3	19.9 ± 2.3	0.654
ODI at final follow-up (%)*	18.4 ± 2.0	18.8 ± 2.1	0.587

* *P* < 0.05 compared with the corresponding preoperative value within the same group. Data were available for all patients at each assessment.

## 4 Discussion

Among the 109 patients with OTLFK included in this study, approximately 69% underwent three-column osteotomy (3CO). Preoperative standing sagittal parameters were broadly comparable between the 3CO and N3CO groups. Postoperative standing radiographs demonstrated that both groups achieved substantial correction, with LKCA, PI–LL mismatch, and TPA values approaching commonly intended alignment targets (LKCA ≈ 10°, PI–LL < 10°, TPA < 20°). The retrospectively calculated Kflx%, which expressed positional reduction relative to the correction ultimately observed, was significantly lower in the 3CO group than in the N3CO group (34.2% vs. 60.6%, *P* < 0.001). Multivariate logistic regression analysis further showed an independent association between Kflx% and the observed implementation of 3CO (OR = 0.940, 95% CI 0.910–0.970). To our knowledge, this is the first study to examine a postoperative target-referenced flexibility metric derived from prone CT scout imaging in relation to 3CO use. The findings describe an association within our institutional practice, but do not establish that Kflx% prospectively predicts whether 3CO is necessary (Figures 5, 6). Both groups also demonstrated postoperative improvement in VAS and ODI during follow-up, providing supportive clinical context for the radiographic findings.

**Figure 5:**
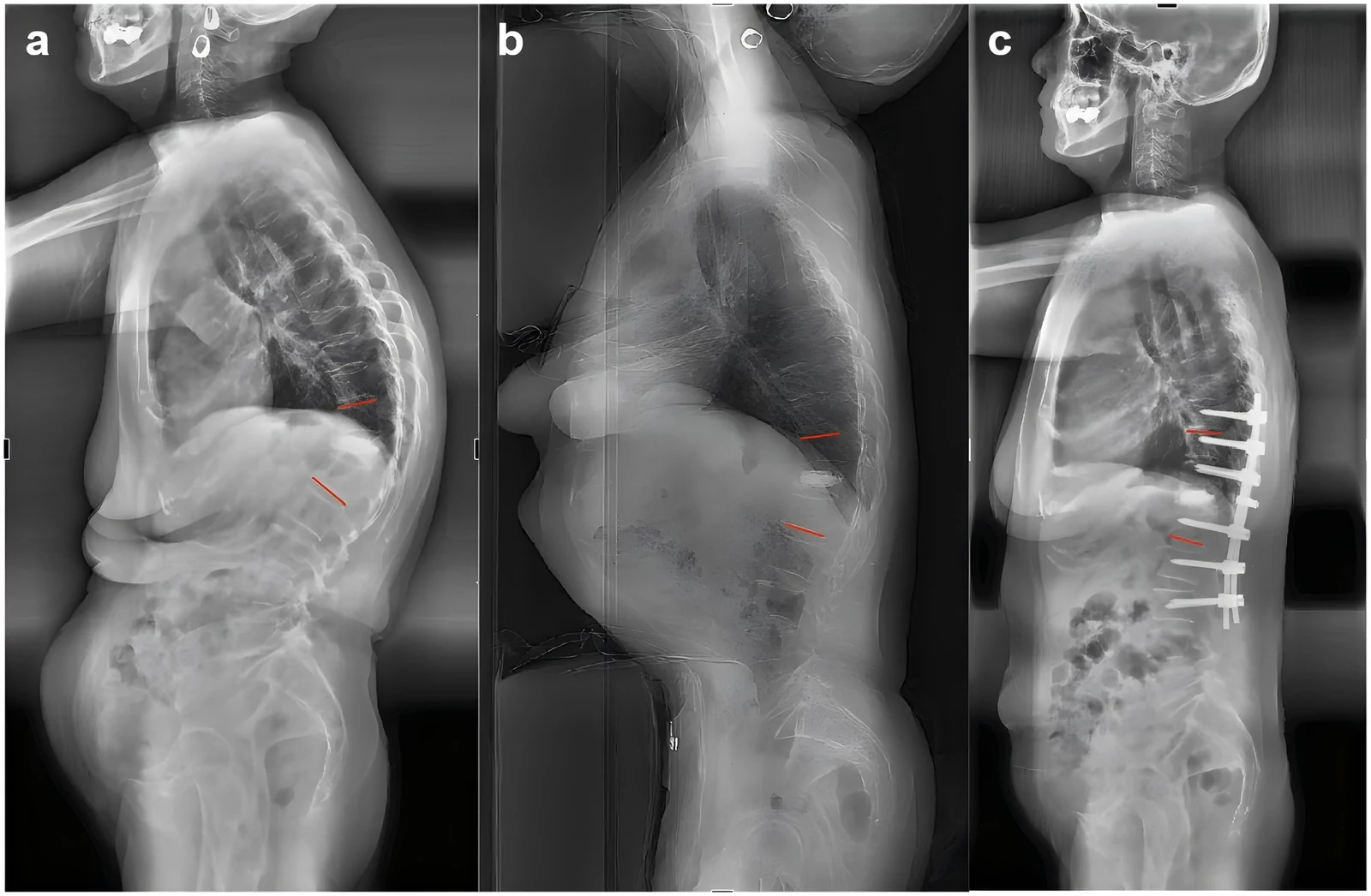
A patient from the N3CO group with a kflx% of 68.9%. (a–c: Preop Standing LKCA 50.7°, Preop Prone LKCA 22.4°, Postop Standing LKCA 9.6°. Correction was achieved by partial resection of the lamina at the T11/12 level.).

**Figure 6:**
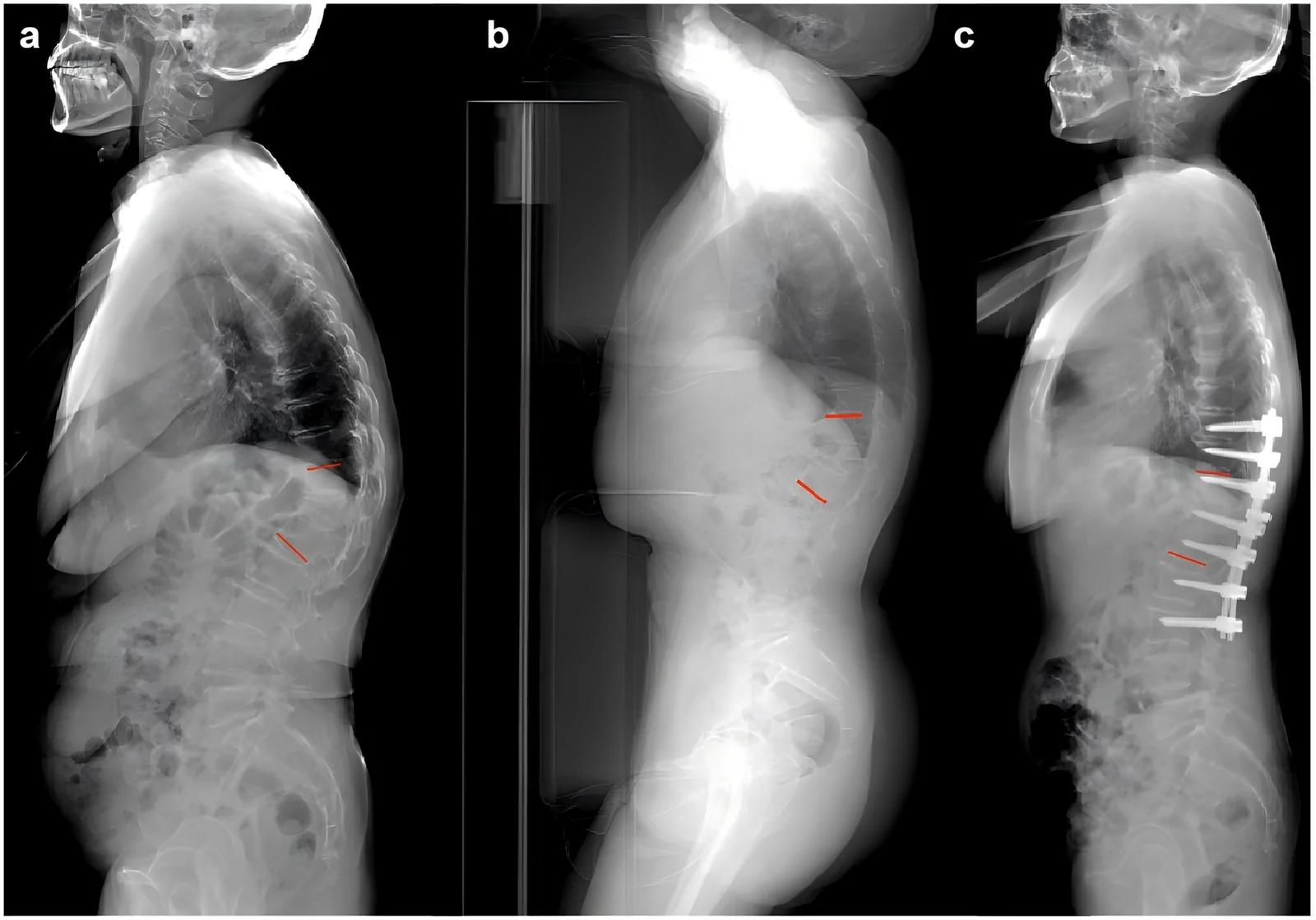
A patient from the 3CO group with a kflx% of 27.0%. (a–c: Preop Standing LKCA 45.1°, Preop Prone LKCA 37.4°, Postop Standing LKCA 16.6°. To achieve adequate correction, a posterior intervertebral osteotomy with anterior column distraction, posterior column compression, and bone graft fusion at the T12/L1 level was performed.).

The formulation of surgical strategies for patients with severe OTLFK is inherently complex, as multiple factors including age, comorbidities, bone quality, surgeon experience, and the severity of kyphosis may play critical roles (21). Among these, assessment of the deformity's relative flexibility is particularly important because it determines whether sufficient correction can be achieved without 3CO. Therefore, comprehensive preoperative evaluation should include both clinical and radiographic assessments of deformity flexibility (11). Our findings are consistent with several recent studies, further confirming the value of positional imaging in preoperative planning for OTLFK (16, 22–25). Wang et al. compared preoperative FLS-CT with intraoperative prone radiographs and found strong agreement in spinal alignment between the two modalities (17). They also demonstrated that prone positioning alone could markedly reduce kyphosis, particularly in patients with moderate to severe deformities, highlighting its significance in sagittal balance restoration. Prone imaging has also been applied to predict proximal junctional kyphosis (PJK) following adult spinal deformity correction. In a study of 31 patients undergoing posterior osteotomy with long-segment fusion, Zhao et al. reported that the thoracolumbar hC7–VSO measured on preoperative FLS-CT, especially at the L2 level, effectively differentiated patients who later developed PJK (18). The authors suggested including T8–L2 hC7–VSO in preoperative risk assessment to identify high-risk individuals and optimize surgical planning and prevention strategies. Similarly, in adult scoliosis surgery, side-bending and traction radiographs are widely used to assess curve flexibility (26). Likewise, supine MRI or prone lateral radiographs can provide valuable information about spinal behavior in unloaded positions (24, 27). Compared with additional dedicated radiographs, the CT scout view can be obtained during routine CT acquisition and may provide a low-dose or dose-comparable adjunct for preoperative positional assessment under optimized protocols (28). Prone positioning facilitates evaluation of the thoracolumbar curvature without the influence of paraspinal muscle antigravity compensation. Future studies should focus on developing more efficient and precise methods to quantify spinal flexibility.

We investigated the relationship between prone-position spinal flexibility and the type of osteotomy performed in patients with different grades of OTLFK (16). Postoperative radiographs showed that patients in the N3CO group achieved 60.6% of their final LKCA correction through intraoperative positioning alone, whereas those in the 3CO group achieved only 34.2%. This finding aligns with previous studies demonstrating that FLS-CT can reproduce the local kyphosis angles observed in the prone position (17, 29). Earlier research has confirmed the importance of prone imaging in evaluating moderate to severe kyphotic deformities associated with chronic osteoporotic thoracolumbar fractures (22, 30). Zhang et al. analyzed 18 patients with severe kyphosis (LKCA > 50°) and examined the relationship between FLS-CT imaging features and osteotomy selection (16). They proposed an imaging-based stratification strategy: when the prone LKCA was < 40° and flexibility exceeded 30%, lower-grade osteotomies or even non-osteotomy fixation could achieve satisfactory correction; conversely, when the prone LKCA remained large and flexibility was insufficient, higher-grade osteotomies were required. This classification highlights the key role of flexibility in determining osteotomy necessity. Liu et al. quantified flexibility in 77 patients with adolescent idiopathic scoliosis (AIS) by calculating the ratio of bending to standing Cobb angles and used restricted cubic spline analysis to identify a threshold of 0.657 (31). When flexibility was < 0.657, Grade II (Ponte) osteotomy achieved greater correction; when flexibility was ≥ 0.657, Grade I (FJO) achieved comparable correction with less blood loss, fewer complications, and shorter hospitalization. Similarly, Karikari et al. examined 70 patients with severe sagittal imbalance (SVA > 10 cm) and compared standing with supine or prone radiographs. They defined flexibility as the percentage increase in lumbar lordosis (LL) from the standing to recumbent position, using the cohort median of 33% as the cutoff to classify patients into flexible (>33%) and stiff (≤33%) groups (32). The results showed that the flexible group achieved substantial sagittal correction regardless of whether 3CO was performed (18 of 35 underwent 3CO), while the stiff group showed limited improvement when 3CO was not used (22 of 35 underwent 3CO). It is noteworthy that flexibility in their study was defined by changes in LL percentage, which represents a fundamental methodological difference from the present study.

Reliable characterization of deformity flexibility is clinically important. However, the present study does not establish a prospective rule for selecting the osteotomy grade. High-grade osteotomies provide strong corrective power. However, they are associated with substantial blood loss and an increased risk of complications. They may also increase the risks of reoperation and instrumentation failure (33). The variation in complication rates across centers may partly reflect differences in preoperative planning and perioperative preparation. In this cohort, a Kflx% below 49.7% had a positive predictive value of 82.9% for the observed use of 3CO. However, Kflx% includes postoperative LKCA. The cutoff was also derived from the same retrospective cohort. Therefore, it should not be regarded as a prospective decision rule. It should not be used alone to recommend or withhold 3CO. Multidisciplinary cooperation can reduce complications and improve outcomes in complex spinal surgery (34, 35). Its effectiveness depends on accurate preoperative planning and adequate team preparation. Surgeons must select an appropriate osteotomy technique and fusion level. They must also determine the most suitable surgical approach. Current correction strategies vary considerably among surgeons (36). A survey of 16 adult spinal deformity cases also showed substantial variation in surgical decisions. Only 41% to 53% of surgeons selected 3CO in some cases. Approximately 50% supported fusion in selected cases (37). Future prospective studies should evaluate fully preoperative flexibility measures. These measures should be assessed using alignment targets defined before surgery. External validation across different institutions and surgeons is required before any threshold can support clinical decision-making in OTLFK.

This study has several limitations. First, Kflx% includes the achieved postoperative standing LKCA in its denominator. It is therefore a postoperative target-referenced metric rather than a purely preoperative predictor. The Kflx% and its 49.7% cutoff were calculated retrospectively. They were not available when the surgical strategy was selected. However, both surgeons had access to the FLS-CT images before surgery. They could consider positional flexibility during surgical planning. This may have introduced incorporation bias. Circularity between the surgical decision and the achieved postoperative correction also cannot be excluded. Therefore, the cutoff only describes the observed practice pattern in this cohort. It should not be interpreted as an indication for 3CO. Second, this was a single-center retrospective study involving two surgeons. Only posterior surgical approaches were included. These factors limit the generalizability of the findings. The two groups also had unequal sample sizes. The 3CO group included 75 patients, whereas the N3CO group included 34 patients. This imbalance may affect the stability of the regression and ROC estimates. Internal resampling and external validation are therefore required. Third, early postoperative radiographic outcomes were available for all patients. However, the study was not designed to assess long-term alignment durability. It also did not primarily evaluate correction loss or proximal junctional kyphosis. Instrumentation-related complications and revision surgery were not evaluated as primary outcomes. Fourth, most patients were women, accounting for 79.8% of the cohort. The mean age was 64.4 years. The mean T-score was −3.0. Therefore, the findings mainly apply to older patients with severe osteoporosis who undergo posterior long-segment correction. The findings should not be directly extrapolated to younger patients or men. They may also not apply to patients with high-energy post-traumatic kyphosis or those treated with other surgical approaches. Prospective multicenter studies are needed. These studies should use alignment targets defined before surgery and include longer follow-up.

## 5 Conclusion

In this retrospective institutional cohort, lower target-referenced Kflx% was associated with the eventual use of 3CO in patients with OTLFK. Because Kflx% incorporated postoperative standing LKCA, it should not be interpreted as a standalone preoperative predictor or as evidence that 3CO was necessary. The 49.7% cutoff is an exploratory within-cohort descriptor and requires prospective validation using alignment targets specified before surgery, internal and external validation, and longer-term follow-up.

## Data Availability

The datasets analyzed during the current study are not publicly available because they contain patient-related clinical and imaging data, but are available from the corresponding authors on reasonable request and subject to institutional approval. Requests to access the datasets should be directed to Haiyang Yu, fy.yhy@163.com.
